# Supplementary material for: Direct costs of blood drawings with pre-analytical errors in tertiary paediatric hospital care
Source: PLoS One. 2023 Aug 25;18(8):e0290636. doi: 10.1371/journal.pone.0290636 (PMC10456202; doi:10.1371/journal.pone.0290636)
Supplement: S1 Table — a: Material consumable cost at the Astrid Lindgren’s Children’s Hospital (2020). b: Material consumables cost and unit use per blood drawing method at Astrid Lindgren’s Children’s Hospital (2020). (DOCX) [file pone.0290636.s001.docx]

| **Supplementary Table 1a.** Material consumables cost at Astrid Lindgren’s Children’s Hospital (2020), Swedish crowns(SEK). | | | |
| --- | --- | --- | --- |
| **Consumables** | **Reference** | **Cost per unit (SEK)** | **Resource use** |
| Venous Micro needle | Sarstetd AB | 7,50 sek | 50/package |
| Venous periphery vein catheter (24G) | BD neoflon | 10,50sek | 50/ package |
| Syringe 5ml | Omnifix | 0,49sek | 100/ package |
| Venous Central lines Vacuum holder + vacuum cannula | BD Vacutainer | 0,15sek + 0,80? (Hittar inte vaccumkanylen I varukatalogen) | 200/ package |
| Venous Butterfly | BD Vacutainer | 6,46sek | 100/ package |
| Venous Vaccutainer  Vacuum holder+ cannula | BD Vacutainer | 0,15sek +0,80sek | 200/ + 480/ package |
|  |  |  |  |
| EMLA(lidokain/prilokain) 5g cream | Aspen Pharma | 79sek | 1/ package |
| Film dressing (4,4x4,4cm) | Tegaderm | 1,81sek | 400/package |
|  |  |  |  |
| Capillary lancet Finger | BD Microtainer | 0,79sek | 200/ package |
| Capillary lancet Heel | Medicarrier Sarstedt Safety-Heel | 11,75sek | 200/ package |
|  |  |  |  |
| Plaster | DermaPlast Kids | 0,16sek | 4000/ package |
| Skin antiseptic (5,x5cm) | Yibon | 0,02sek | 13500/ package |
| Surgical tape | Micropore | 2,99sek | 240/ package |
| Micro tube MAP 0,5ml | BD EDTA MAP | 4,20sek | 200/ package |
| Micro tube (0,5ml) | BD Li-hep | 1,89sek | 200/ package |
| Vacuum Tube (3-5ml). | BD EDTA | 0,75sek | 100/ package |
| Injection membrane | Bionector | 4,75sek | 2400/ package |
|  |  |  |  |
| Infusion connector (3 way) | Sendal | 4,64sek | 200/ package |

| **Supplementary Table 1b.** Material consumables cost and unit use per sampling method at Astrid Lindgren’s Children’s Hospital (2020), Swedish crons(SEK). | | | | |
| --- | --- | --- | --- | --- |
| **Blood draw method** | **Category** | **Unit use per drawing** | **Units cost/time per drawing** | **Cost per blood draw -summery** |
| Venous blood drawing  “Micro needle” | Materials costs per piece | 1p Micro needle  2p Swabs  1p Plaster  1p Microtube  2g EMLAcream | 1x 7,50 sek  2x 0,02 sek  1x 0,16 sek  1x 3, 05 sek  1x 33,41sek | 44,16sek |
| Venous blood drawing  “Venous periphery vein catheter (24G) (New insertion)” | Materials costs per piece | 1p Pvc  3p Syringe(5ml)  2p Swabs  1p Microtube  2g EMLAcream | 1x 10,50 sek  3x 0,49 sek  2x 0,02 sek  1x 3,05 sek  1x 33,41sek | 48,47sek |
| Venous blood drawing  “Venous periphery vein catheter (24G) (Draw) | Materials costs per piece | 3p Syringe 5ml  2p Swabs  1p Microtube  1p 3-way connector | 3x 0,49 sek  2 x 0,02 sek  1x 3,05 sek  1x 4,64sek | 9,2sek |
| Venous blood drawing  “Butterfly needle” | Materials costs per piece | 1p Butterfly needle  2p Swabs  1 Microtube  1p Plaster  2g EMLAcream | 1x 6,46 sek  2x 0,02 sek  1x 3,05 sek  1x 0,16 sek  1x 33,41sek | 43,12sek |
| Venous blood drawing  “Straight needle Vaccutainer” | Materials costs per piece | 1p Vacuum holder  1p Straight needle  2p Swabs  1p Plaster  1p Microtube  2g EMLAcream | 1x 0,15sek  1x 0,80sek  2x 0,02 sek  1x 0,16 sek  1x 3,05 sek  1x 33,41sek | 37,61sek |
| Venous blood drawing  “Central venous Line draw” | Materials costs per piece | 1p Vacuum holder  1p Vacuum cannula  2p Swabs  3p Syringe 10ml  1p Microtube  1p injektionsmemb  1p 3-way connector | 1x 0,15sek  1x 0,80sek  2x 0,02 sek  3x 0,49 sek  1x 3,05 sek  1 x 4,75sek  1x 4,64sek | 14,9sek |
| Capillary blood draw  “Finger punction” | Materials costs per piece | 1p Finger lancet  2p Swabs  1p Plaster  1p Microtube | 1 x 0,79 sek  2x 0,02 sek  1x 0,16 sek  1x 3,05 sek | 4,04sek |
| Capillary blood draw  “Heel side punction” | Materials costs per piece | 1p heel lancet  2p Swabs  1p Plaster  1p Microtube | 1 x 11,75sek  2x 0,02 sek  1x 0,16 sek  1x 3,05 sek | 15sek |
