# Supplementary material for: Direct costs of blood drawings with pre-analytical errors in tertiary paediatric hospital care
Source: PLoS One. 2023 Aug 25;18(8):e0290636. doi: 10.1371/journal.pone.0290636 (PMC10456202; doi:10.1371/journal.pone.0290636)
Supplement: S2 Table — (DOCX) [file pone.0290636.s002.docx]

| **Supplementary Table 2: The cost of blood analyses of blood ordered and analysed to the chemistry, haematology and coagulation section at Astrid Children’s Lindgren’s Hospital 2019.** | | | | |
| --- | --- | --- | --- | --- |
| **Section** | **Analyses** | **Cost per unit/SEK** | **Total number of analyses** | **Total cost per year /SEK** |
| Chemistry | P-CRP | 11,13 | 38 030 | 423 382,23 |
|  | P-Kreatinin | 5,56 | 35 924 | 199 788,24 |
|  | P-Natrium | 5,56 | 22 964 | 127 711,51 |
|  | P-Kalium | 5,56 | 22 563 | 125 481,69 |
|  | P-Albumin | 5,56 | 23 071 | 128 307,05 |
|  | P-Bilirubin | 5,56 | 18 583 | 103 346,99 |
|  | P-ASAT | 5,56 | 22 009 | 122 404,48 |
|  | P-ALAT | 5,56 | 25 143 | 139 836,33 |
|  | P-Fosfat | 5,56 | 15 605 | 86 783,49 |
|  | P -Magnesium | 11,13 | 13 885 | 154 580,99 |
|  | P-Calcium | 5,56 | 14 606 | 81 228,49 |
|  | P-Bilirubin,konj | 5,56 | 8 612 | 47 891,92 |
|  | P-Urea | 5,56 | 8 630 | 47 992,29 |
|  | P-GT | 5,56 | 10 197 | 56 709,78 |
|  | P-LD | 5,56 | 5 683 | 31 602,63 |
|  | fP-Triglycerid | 5,56 | 3 706 | 20 610,93 |
|  | P-Klorid | 30,57 | 2 351 | 71 858,62 |
|  | P-Urat | 5,56 | 3 874 | 21 544,80 |
|  | P-ALP (Alk fosfatas) | 5,56 | 2 118 | 11 779,99 |
|  | P-Glukos | 5,56 | 4 542 | 25 263,07 |
|  | P-Troponin, högkänsligt | 79,12 | 1 789 | 141 542,38 |
|  | P-Pankreasamylas | 41,01 | 4 396 | 180 277,45 |
|  | P-Cystatin C | 24,15 | 5 537 | 133 739,80 |
|  | P-NT-proBNP | 233,10 | 1 218 | 283 911,24 |
|  | P-Järn | 5,56 | 2 568 | 14 283,29 |
|  | P-Methotrexat | 869,64 | 1 123 | 976 601,50 |
|  | S-Procalcitonin | 244,86 | 5 363 | 1 313 169,59 |
|  | S-Ferritin (ModE) | 44,52 | 4 755 | 211 677,51 |
|  |  |  |  |  |
| Hematology | B-Blodstatus | 18,97 | 58 813 | 1 115 920,48 |
|  | B-Celler | 24,15 | 35 923 | 867 602,19 |
|  | B-Retikulocyter | 36,89 | 4 260 | 157 142,97 |
|  | B-SR | 22,92 | 6 126 | 140 403,37 |
|  | B-Standardbikarbonat | 66,83 | 1 273 | 85 080,20 |
|  |  |  |  |  |
| Coagulation | P-PK(INR) | 33,38 | 11 734 | 391 629,89 |
|  | P-APT-tid | 55,64 | 8 044 | 447 557,43 |
|  | P-Fibrinogen (koag) | 74,80 | 6 234 | 466 302,28 |
|  | P-Fibrin-D-dimer | 151,20 | 2 192 | 331 421,74 |
|  | P-Antitrombin (enz) | 129,10 | 2 786 | 359 659,06 |
|  | P FIBRIN, LÖSLIGT | 284,74 | 2 003 | 570 337,98 |
| Total cost per analyses: |  | 21,8 | 468 233 | 10 216 365 |
| Cost per analyses: 10216365sek / 468233analyses = 21,8SEK/analyses=2,06Euro | | | | |
