# Supplementary material for: Direct costs of blood drawings with pre-analytical errors in tertiary paediatric hospital care
Source: PLoS One. 2023 Aug 25;18(8):e0290636. doi: 10.1371/journal.pone.0290636 (PMC10456202; doi:10.1371/journal.pone.0290636)
Supplement: S3 Table — (DOCX) [file pone.0290636.s003.docx]

| **Supporting information 3:  Observation protocol,** Direct costs associated with failed blood sample collections in tertiary paediatric hospital care |
| --- |

| **Supplementary Table 3.1: Clinical Observations 17 total (7 capillary, 10 venous), in May 2021 at Astrid Lindgren’s Children’s Hospital** | | | | | | | | | | | | |  |
| --- | --- | --- | --- | --- | --- | --- | --- | --- | --- | --- | --- | --- | --- |
| **Time observations - variables** | | | | | | | **Results** | | | | | |  |
| N= Samplings | Place:  Ward/ ED | Sampling type | Personnel  Reg Nurse = 1  Nurse Ass =2 | Age | Type of analyses | N of tubes | Time before sampling  (ordination, material) | Time during sampling  (in the room) | Time after sampling (go to the pneumatic tube machine and send in the computer) | Time sampling total (from preparing to sending in the computer) | Time waiting the results  (from sending to getting the results) | PAE  YES =1  NO = 2 | |
| 1 | ED | Capillary | 2 | 2yr 10m | Crea | 1 | 2+5min | 10min | 3min | 18min | 33min | 2 | |
| 2 | ED | Venous | 1+2 | 5yr | CBC, CRP, SR | 2 | 0930 2min, 5min | 1157-1209 12min | 1216 7min | 26min | 1235delsvar,1302,fullsvar  46min | 2 | |
| 3 | ED | Venous | 1+2 | 6yr 3m | CBC, CRP | 2 | (2+5min) 7min | 12min | 3min | 22min | 29min | 2 | |
| 4 | ED | Capillary | 1 | 13m (premature) | CBC diff, CRP, | 2 | (2min+5min) 7min | 5min | 2min | 14min | 34min | 2 | |
| 5 | ED | Venous | 1+2 | 10yr | CBC, CRP | 2 | (2min+2min) 4min | 8min | 3min | 15min | 120min | 2 | |
| 6 | ED | Capillary | 2 | 3yr 9m | CBC, diff, CRP | 2 | (2min + 2min)  4min | 15min | 3min | 22min | 46min | 1 | |
| 7 | ED | Venous | 1+2 | 3yr 5m | Prokalcitonin, CBC, CRP-elstatus | 3rör | 10min | 16min | 4min | 30min | 50min | 2 | |
| 8 | ED | Capillary | 1+ student | 3yr 2m | CBC, bloodgas, CRP | 1rör + 2st kassett | 2min+3min | 5min | 5min (PNA) 9min | 19min | 5min (PNA)  25min | 2 | |
| 9 | Ward 10 | Venous | 1+2 | 12yr | APT, Fibrin D Dimer | 1rör | 5min | 15min | 2min | 22min | 50min | 2 | |
| 10 | Ward 10 | Venous | 1+2 | 1m | CRP, krea, CBC | 2rör | 10min | 25min | 2min | 37min | 68min | 2 | |
| 11 | Ward 10 | Venous | 1+2 | 13yr | CRP, SR, LPK | 2rör | 10min | 10min | 2min | 22min | 173min | 2 | |
| 12 | Ward 10 | Capillary | 1+2 | 10yr | CBC, CRP | 2rör | 5min | 10min | 2min | 17min | 120min | 1 | |
| 13 | Ward 10 | Venous | 1 | 1yr 11mån | CBC, coagulationstatus. Crp etc | 5rör | 10min | 8min | 2min | 22min | 62min | 2 | |
| 14 | Ward 10 | Venous | 1 | 3yr 4m | CBC, liver-status, CRP | 2rör | 12min | 6min | 5min | 23min | 52min | 2 | |
| 15 | Ward 10 | Venous | 1 | 9yr 3m | CBC, Elstatus | 2rör | 5min | 8min | 3min | 16min | 45min | 2 | |
| 16 | Ward 10 | Capillary | 1+2 | 2m | CBC | 1rör | 10min | 8min | 4min | 22min | 25min | 2 | |
| 17 | Ward 10 | Capillry | 1+2 | 7yr 4m | CBC,CRP | 2rör | 6min | 7min | 2min | 15min | 65min | 2 | |

| **Supplementary Table 3.2: Clinical Observations 17 total (7 capillary, 10 venous), in May 2021 at Astrid Lindgren’s Children’s Hospital -summarized** | | | |
| --- | --- | --- | --- |
| Time before sampling | 5, 5, 5, 2, 4, 2, 10, 3, 5,10,10,5, 10,12,5,10,6 =109min | Min: 2min  Max: 12min | Mean: 109/17= 6,4min |
| Time during sampling | 10, 12,12,5,8,15,16,5,15,25,10, 10,8,6,8,8,7 = 180min | Min: 5min  Max: 25min | Mean: 180/17 = 10,6min |
| Total sampling process | 18,26,22,14,15,22,30,19,22,37, 22,17,22,23,16,22,15 = 362min | Min: 14min  Max: 37min | Mean: 362/17 = 21,3min |
| Waiting for results | 33,46,29,34,120,46,50,25,50,68, 173,120,62,52,45,25,65 = 1043min | Min: 25min Max: 173min | Mean: 1043/17= 61,4min |
| Total testing process |  |  | 21,3+61,4min =  82,7min |
