# Supplementary material for: Direct costs of blood drawings with pre-analytical errors in tertiary paediatric hospital care
Source: PLoS One. 2023 Aug 25;18(8):e0290636. doi: 10.1371/journal.pone.0290636 (PMC10456202; doi:10.1371/journal.pone.0290636)
Supplement: S4 Table — (DOCX) [file pone.0290636.s004.docx]

**Supplementary information 5:** Personnel salaries

| **Personnel category** | **Average salary per hours (SEK)** | **Average salary per month**  **(SEK)*** |
| --- | --- | --- |
| Reg Nurse Children’s ward | 238 | 38012 |
| Reg Nurse Neonatal ward | 233 | 37268 |
| Reg Nurse Intensive care | 259 | 41434 |
| Nurse assistent child ward | 173 | 27722 |
| Nurse assistent neo ward | 172 | 27456 |
| Nurse assistent intensive | 160 | 29842 |
| Medical doctors child ward | 404 | 64630 |
| Medical doctors neo ward | 426 | 68136 |
| Medical doctors intensive | 486 | 77729 |

*Data from hospital economic information system, Tableau software©
