# Supplementary figures and images for: Direct costs of blood drawings with pre-analytical errors in tertiary paediatric hospital care
Source: PLoS One. 2023 Aug 25;18(8):e0290636. doi: 10.1371/journal.pone.0290636 (PMC10456202; doi:10.1371/journal.pone.0290636)

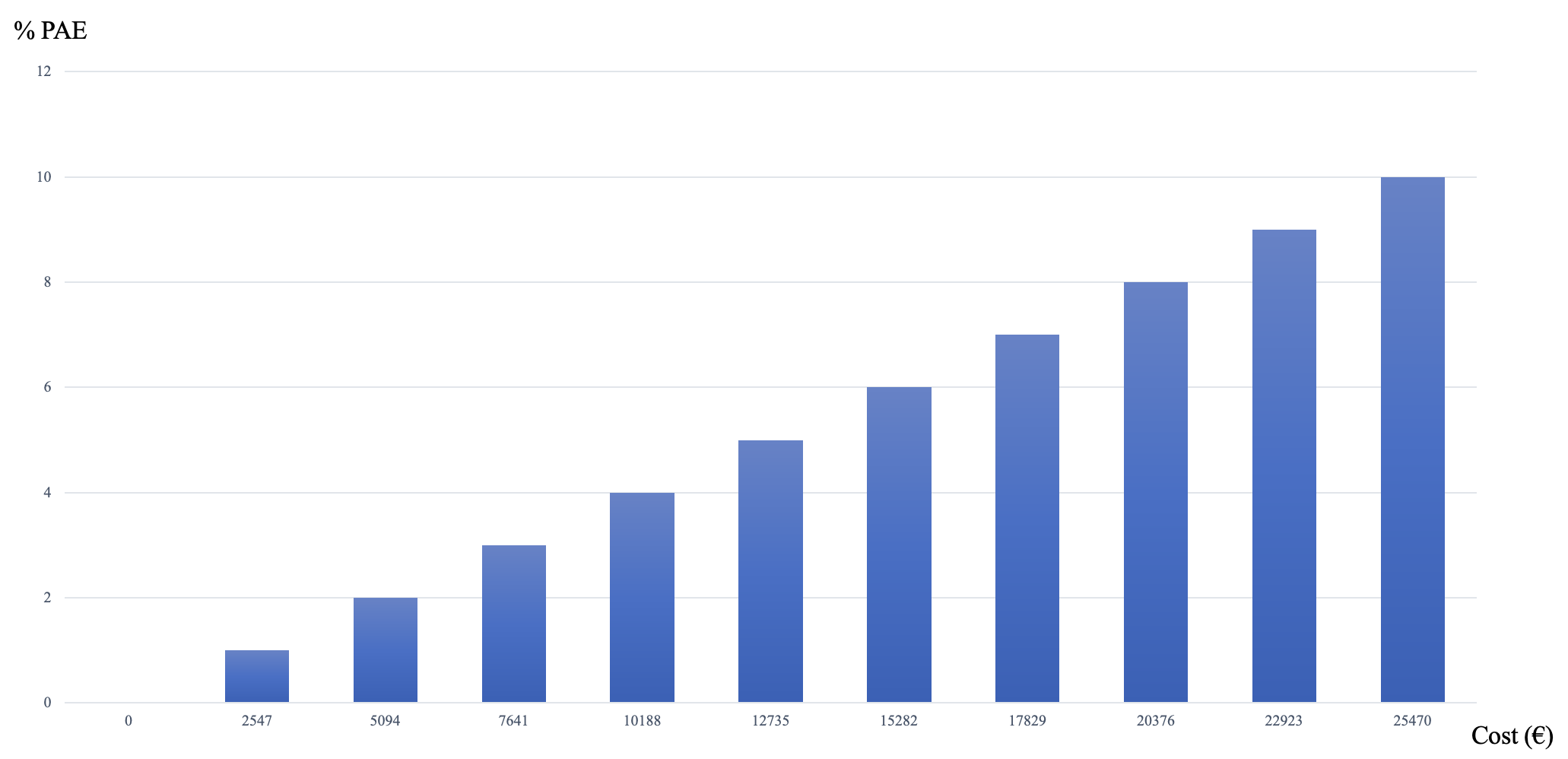

Supplement: S1 Fig — (TIFF) [file pone.0290636.s005.tiff]
