# Supplementary material for: Direct costs of blood drawings with pre-analytical errors in tertiary paediatric hospital care
Source: PLoS One. 2023 Aug 25;18(8):e0290636. doi: 10.1371/journal.pone.0290636 (PMC10456202; doi:10.1371/journal.pone.0290636)
Supplement: Attachment — Submitted filename: reply to the reviewers.docx [file pone.0290636.s006.docx]

Dear Editor,

Thank you for letting us revise our manuscript ”Direct costs associated with failed blood sample collections in tertiary paediatric hospital care” for publication in PLOS ONE.

We have answered point by point the questions aroused from the original review.

Journal Requirements:

When submitting your revision, we need you to address these additional requirements.

1. Please ensure that your manuscript meets PLOS ONE's style requirements, including those for file naming. The PLOS ONE style templates can be found at

<https://journals.plos.org/plosone/s/file?id=wjVg/PLOSOne_formatting_sample_main_body.pdf>and

[https://journals.plos.org/plosone/s/file?id=ba62/PLOSOne_formatting_sample_title_authors_affiliations.pdf](https://eur01.safelinks.protection.outlook.com/?url=https%3A%2F%2Fjournals.plos.org%2Fplosone%2Fs%2Ffile%3Fid%3Dba62%2FPLOSOne_formatting_sample_title_authors_affiliations.pdf&data=05%7C01%7Chenrik.hjelmgren%40ki.se%7C352adc055d304419f08708daeea7bf75%7Cbff7eef1cf4b4f32be3da1dda043c05d%7C0%7C0%7C638084702059592672%7CUnknown%7CTWFpbGZsb3d8eyJWIjoiMC4wLjAwMDAiLCJQIjoiV2luMzIiLCJBTiI6Ik1haWwiLCJXVCI6Mn0%3D%7C3000%7C%7C%7C&sdata=pESinXnhF6%2Faw8pUqNXjvxU6yqjvwrVI4gdBPBGbsQI%3D&reserved=0)

- Reply: Thanks. We have revised accordingly.

2. Please provide additional details regarding participant consent. In the ethics statement in the Methods and online submission information, please ensure that you have specified what type you obtained (for instance, written or verbal, and if verbal, how it was documented and witnessed). If your study included minors, state whether you obtained consent from parents or guardians. If the need for consent was waived by the ethics committee, please include this information.

- Reply: Thank you for pointing this out. We have added details regarding participants consent information in the manuscript under ethical consideration.

3. You indicated that ethical approval was not necessary for your study. We understand that the framework for ethical oversight requirements for studies of this type may differ depending on the setting and we would appreciate some further clarification regarding your research. Could you please provide further details on why your study is exempt from the need for approval and confirmation from your institutional review board or research ethics committee (e.g., in the form of a letter or email correspondence) that ethics review was not necessary for this study? Please include a copy of the correspondence as an "Other" file.

- Reply: Thank you for this question. The study was exempted from ethical review due to the following quote from the ethical committee. *“The research referred to in the application for change does not involve any processing of sensitive personal data. Nor is the research otherwise of such a nature that it is covered by the Act (2003:460) on ethical review of research involving humans. The ethics review authority cannot therefore take up the application for change for consideration for approval.”*

Nevertheless, we as researchers have taken ethical considerations in mind in all this study process according to regulations in the Helsinki declaration.”

4. In your Data Availability statement, you have not specified where the minimal data set underlying the results described in your manuscript can be found. PLOS defines a study's minimal data set as the underlying data used to reach the conclusions drawn in the manuscript and any additional data required to replicate the reported study findings in their entirety. All PLOS journals require that the minimal data set be made fully available. For more information about our data policy, please see <http://journals.plos.org/plosone/s/data-availability>.

Upon re-submitting your revised manuscript, please upload your study’s minimal underlying data set as either Supporting Information files or to a stable, public repository and include the relevant URLs, DOIs, or accession numbers within your revised cover letter. For a list of acceptable repositories, please see <http://journals.plos.org/plosone/s/data-availability#loc-recommended-repositories>. Any potentially identifying patient information must be fully anonymized.

Important: If there are ethical or legal restrictions to sharing your data publicly, please explain these restrictions in detail. Please see our guidelines for more information on what we consider unacceptable restrictions to publicly sharing data: <http://journals.plos.org/plosone/s/data-availability#loc-unacceptable-data-access-restrictions>. Note that it is not acceptable for the authors to be the sole named individuals responsible for ensuring data access.

We will update your Data Availability statement to reflect the information you provide in your cover letter.

- Reply: The original data set from Flexlab cannot be shared as it carries personnel data, which is regulated by our the ethical approval (DNR 2021-00846), see under Ethical Considerations, page 8. However, the observation protocol can be published, see supporting information files 3.

5. We note that you have stated that you will provide repository information for your data at acceptance. Should your manuscript be accepted for publication, we will hold it until you provide the relevant accession numbers or DOIs necessary to access your data. If you wish to make changes to your Data Availability statement, please describe these changes in your cover letter and we will update your Data Availability statement to reflect the information you provide.

- Reply: Thank you. We believe we answered this above.

6. We noted in your submission details that a portion of your manuscript may have been presented or published elsewhere. Please clarify whether this publication was peer-reviewed and formally published. If this work was previously peer-reviewed and published, in the cover letter please provide the reason that this work does not constitute dual publication and should be included in the current manuscript.

- Reply: This study has not been published in a scientific paper elsewhere, but data from the study is included in corresponding author Henrik Hjelmgren’s doctoral thesis, defended in February 2022 and published in open archive at Karolinska Institutet <https://openarchive.ki.se/xmlui/bitstream/handle/10616/47834/Thesis_Henrik_Hjelmgren.pdf?sequence=1&isAllowed=y>

7. Please include captions for your Supporting Information files at the end of your manuscript, and update any in-text citations to match accordingly. Please see our Supporting Information guidelines for more information: <http://journals.plos.org/plosone/s/supporting-information>.

- Reply: Thank you. We revised accordingly.

**Comments to the Author**

**Reviewer #1**:

The authors present important data for cost estimation of preanalytical errors, which could serve as basis for negotiations with hospital management, aiming to improve quality of care.
The authors emphasise that their study is the first one focusing on paediatric care.
If focusing only on children, the PAE rate from the laboratory has to be recalculated, including only samples from pediatric care.
- Reply: Thank you for your comment. The PAE rate includes only samples from pediatric care. Please see further information in “Hjelmgren H, et al. Retrospective study showed that blood sampling errors risked children's well-being and safety in a Swedish paediatric tertiary care. Acta Paediatr, 2019. 108(3): p. 522-528”

How was the total allocated time to the blood sampling procedures calculated/observed? If observations were done, how many per profession?
- Reply: Thank you for your question. The total allocated time which was observed and calculated are presented in the timesheet which is summarised in S3 Table and now added details in S4. We made 17 observations whereby 15nurses and 12 nurse assistants were included. 10 times there were both a nurse and a nurse assistant. Just a few medical doctors were observed in the beginning of the study to estimate the average 5min time which was not documented in the protocol but with memos. We clarified this and added in the method section of the manuscript under personnel cost.

30 minutes for blood collection for a nurse seems quite long, especially when including 15 minutes nurse assistant and 5 min doctors time
- Reply: Thank you for pointing this out. The observations included preparations, taking the sample, as well as the things you need to do after an sampling. We added to Supporting information 3 a more detailed observation protocol to be able to clarify for the reader our time observations. It’s possible so that inward hospital settings the time can take longer than outpatient clinics as the HCPs has many different tasks going on at the same time.

Why are personell costs added up? Same for material costs. I believe that not all types of needles are used simultaneously for each blood collection
- Reply: Thank you for this question. To align with to use of different materials for blood sampling the costs were added up based on the study “Capillary blood sampling increases the risk of preanalytical errors in pediatric hospital care: Observational clinical study” showing the prevalence of different blood sampling methods inward at Astrid Lindgren Children’s Hospital. The added up personal costs reflects the different healthcare personnel’s inward that are involved in the blood sampling process, see revised Table 1.

Fig 3 needs further explanation as well as an x-axis title. The figure needs to be understandable only by the image and the legend.
- Reply: Thank you for pointing this out. We have clarified the x-axis title and put the figure 3 to supplementary information 4 instead.

**Reviewer #2:** Occasional grammatical errors
The paper should state at the outset that the paper is an analysis of data collected at different times (PAE data from 2013/2014, finance data from other years etc). This is not unreasonable but should be described as the structure for the work.
- Reply: Thank you for this suggestion. We have added this to the method/design section at page 4.

The procedure for the collection of data for personnel cost etc by observation should be described in the methods.
- Reply: Thank you for this comment. We revised and clarified in the manuscript under personnel cost in the methods section.

The discussion mentions the use of standard protocols to reduce bias and this should be in the methods.
- Reply: Thank you for this comment. It was not a “standard” protocol it was an observation protocol adjusted for this purpose only. We have now added a more detailed information in the S3 table - observation protocol. We also clarified the same information under the method section personnel cost.

The 'hospitalisation cost' is unclear. I assumed this was the overheads costs (building, utilities, service costs etc) but the figure attached includes the steps of the pre, analytical and post phases.
- Reply: Thank you for mention this point. The hospitalization cost is as you say overhead costs but only during the time sampling occurred (pre to-post phases). We revised and made a clarification under this method section page 7 and row 151.

Table 1 would benefit from clarification - the first column (allocation per blood sampling) has data in minutes, % etc. with little explanation.
- Reply: Thanks for your comment, Table 1 has been revised accordingly.

The discussion reviews data from other papers and attempts to explain the variations. The overall variation between papers is enormous=: this may be because the data has been collected so differently that it is not comparable. I wonder if there are any components between the different studies that can be normalised to make them comparable?
- Reply: Thank you for this comment. We totally agree with you and it was a stress on how to attempt to refer to other publications in this field. Our attempt was to normalize and compare with the frequency 10,000samplings and by converting and summarize the cost in euros.

The histogram figure presented is difficult to understand, even when referring to the legend in the text. It requires revising to contribute to the paper. In fact, all 3 figures would benefit from refining.
- Reply: Thank you for this point. We refined Fig 1 and Fig 3. We moved Fig 3 to Supplementary.

**Reviewer #3:** This manuscript describes a calculation over the costs associated with blood sample collections that due to preanalytical errors must be repeated. The study is based on register data from 2013-2014 and the data on the preanalytical errors has previously been published. Although I find it relevant to calculate the expenses caused by errors in the blood sampling, I do not find the study merits a scientific report. The novelty is too meager, and the basic findings are already published, If any, these calculations should have been a part of the first paper. Also, many things have changed in awareness on the preanalytical area in the last ten years, especially in the pediatric field.
-Reply: Thank you for your remark. To the best of our knowledge we believe that there is novelty and scientific awareness in the study which could be of interest to the readers of PLOS1 as well as clinicians around the world.

Apart from this major remark, I have a number of minor remarks:

I do not understand why there are no phlebotomists involved in the blood sampling: In many countries, a subgroup of phlebotomists are specially trained in pediatric sampling, which has been shown to reduce the number of preanalytical errors. The authors should comment on this.
- Reply: Thank you for pointing this out. This is the case in some countries as you mention but not in the Swedish context. Especially when it comes to inward and hospital settings phlebotomists are rare. In the paediatric field there are several advantages if the staff know their patients and has knowledge on children’s background, different development stages as well as medical conditions. This is seldom anything phlebotomist have time and knowledge in which is something to debate of course. To our knowledge we haven’t seen any studies/publication comparing peadiatric draws by phlebotomist and peadiatric draws by peadiatric nurses with the outcome of PAE and children’s satisfaction and costs. We added in the manuscript discussion part Page 16, row 255:

“Another possibility to reduce cost is the use of phlebotomy teams [29]. In the Swedish context, phlebotomists only work in outpatient clinics, and to the best of our knowledge, phlebotomy teams have not been investigated in a paediatric in-ward hospital setting.

When the numbers of blood samplings are counted, there is a confusion between the blood sampling process and the number of samples. I would imagine that the 50,040 samples drawn are from are far lesser number of blood samplings; in general, 3-5 samples are taken per drawing, and the number of drawings are therefore only around 15,000. This alters the calculations, which are made per blood sample, which probably more correctly depicts a price per sampling (and 3-5 times more blood samples).
- Reply: Sorry for the misunderstanding. While the number of specific blood analyses were 1,148,716, for the two years 2013-2014, the number of unique blood samplings were 108,080. We then calculated the annual mean to 54,040 (Hjelmgren et al 2019).

When calculating the prices, I wonder were costs for blood tubes are incorporated. Please specify.
- Reply: Thank you for this question. This was incorporated in the material cost of each sampling method depending on the method. Supplementary table 1 shows the different tubes cost which was used.

In the original paper from 2019, it is shown that the top 2 in preanalytical errors are clotted samples (51,3%) and incorrectly filled samples (23,3%). The latter is actually only a real problem in terms of coagulation testing, and the pricing therefore depends on the analysis requested, as probably only few needs a re-draw.
- Reply: Thank you for this comment, however we don’t agree. Incorrect filled samples can occur in all different testings. In our study from 2019 we found the number of incorrect filled samples in 7494 analyses in the hematology section and 1225 analyses in the coagulation and 4230 analyses in the chemistry section. But by proportion of the blood analyses taken, coagulation had 2% and hematology and chemistry had 1% respectively. Due to importance of the clinical value of coagulation testing this is probably to most common re-draw analysis in paeditrics as the filling is to be on the exact marking on the tube as well.

I find that Figure 1-3 is irrelevant and can be omitted.
- Reply: ok. Thank you for the suggestion. We omitted figure 3 to supplementary information but kept figure 1- 2 in the manuscript but made small adjustments.

6. PLOS authors have the option to publish the peer review history of their article ([what does this mean?](https://eur01.safelinks.protection.outlook.com/?url=https%3A%2F%2Fjournals.plos.org%2Fplosone%2Fs%2Feditorial-and-peer-review-process%23loc-peer-review-history&data=05%7C01%7Chenrik.hjelmgren%40ki.se%7C352adc055d304419f08708daeea7bf75%7Cbff7eef1cf4b4f32be3da1dda043c05d%7C0%7C0%7C638084702059592672%7CUnknown%7CTWFpbGZsb3d8eyJWIjoiMC4wLjAwMDAiLCJQIjoiV2luMzIiLCJBTiI6Ik1haWwiLCJXVCI6Mn0%3D%7C3000%7C%7C%7C&sdata=vhyPDkGa1wH853%2BZt%2FzYHys%2BHefVK3CXAGhfIt8ff4Q%3D&reserved=0)). If published, this will include your full peer review and any attached files.


If you choose “no”, your identity will remain anonymous but your review may still be made public.


**Do you want your identity to be public for this peer review?** For information about this choice, including consent withdrawal, please see our [Privacy Policy](https://eur01.safelinks.protection.outlook.com/?url=https%3A%2F%2Fwww.plos.org%2Fprivacy-policy&data=05%7C01%7Chenrik.hjelmgren%40ki.se%7C352adc055d304419f08708daeea7bf75%7Cbff7eef1cf4b4f32be3da1dda043c05d%7C0%7C0%7C638084702059592672%7CUnknown%7CTWFpbGZsb3d8eyJWIjoiMC4wLjAwMDAiLCJQIjoiV2luMzIiLCJBTiI6Ik1haWwiLCJXVCI6Mn0%3D%7C3000%7C%7C%7C&sdata=MZI8VCHn6SMZVCheS%2Fb%2FS3eFP9GSO2Lv67ZGvYBhERc%3D&reserved=0).

Reviewer #1: No

Reviewer #2: No

Reviewer #3: No
