# Supplementary material for: Direct costs of blood drawings with pre-analytical errors in tertiary paediatric hospital care
Source: PLoS One. 2023 Aug 25;18(8):e0290636. doi: 10.1371/journal.pone.0290636 (PMC10456202; doi:10.1371/journal.pone.0290636)
Supplement: Attachment — Submitted filename: RESPONSE TO REVIEWERS.docx [file pone.0290636.s007.docx]

RESPONSE LETTER #2

PONE-D-22-29412R1

Direct costs associated with failed blood sample collections in tertiary paediatric hospital care

PLOS ONE

Dear Dr. Hjelmgren,

Thank you for submitting your manuscript to PLOS ONE. After careful consideration, we feel that it has merit but does not fully meet PLOS ONE’s publication criteria as it currently stands. Therefore, we invite you to submit a revised version of the manuscript that addresses the points raised during the review process.

Please submit your revised manuscript by **Jul 29 2023 11:59PM.** If you will need more time than this to complete your revisions, please reply to this message or contact the journal office at [plosone@plos.org](mailto:plosone@plos.org). When you're ready to submit your revision, log on to <https://www.editorialmanager.com/pone/> and select the 'Submissions Needing Revision' folder to locate your manuscript file.

Please include the following items when submitting your revised manuscript:

- A rebuttal letter that responds to each point raised by the academic editor and reviewer(s). You should upload this letter as a separate file labeled 'Response to Reviewers'. YES.
- A marked-up copy of your manuscript that highlights changes made to the original version. You should upload this as a separate file labeled 'Revised Manuscript with Track Changes'. YES.
- An unmarked version of your revised paper without tracked changes. You should upload this as a separate file labeled 'Manuscript'. YES.

We look forward to receiving your revised manuscript.

Kind regards,

**Janne Cadamuro**
Guest Editor
PLOS ONE

Journal Requirements:

Please review your reference list to ensure that it is complete and correct. If you have cited papers that have been retracted, please include the rationale for doing so in the manuscript text, or remove these references and replace them with relevant current references. Any changes to the reference list should be mentioned in the rebuttal letter that accompanies your revised manuscript. If you need to cite a retracted article, indicate the article’s retracted status in the References list and also include a citation and full reference for the retraction notice.

Reply:
Dear Editor Dr Janne Cadamuro and reviewers. We sincerely thank you for the opportunity to resubmit a revised and improved version of the manuscript. We are grateful for the time and efforts you and the reviewer spent to provide valuable feedback. We have considered all comments by answering the comments point by point below in red, and we have revised the manuscript accordingly. In addition, we have changed the original title “*Direct costs associated with failed blood sample collection in tertiary paediatric hospital care”* to, in our opinion more informative and balanced summary, “*Direct costs of blood drawings with pre-analytical errors in tertiary paediatric hospital care*”.

These are the changes in the new reference list:

Number 20: Inserted reference:
20.	*Piazza J, Merkel S, Neusius H, Murphy S, Gargaro J, Rothberg B, et al. It's not just a needlestick: exploring phlebotomists' knowledge, training, and use of comfort measures in pediatric care to improve the patient experience. J Appl Lab Med. 2019;3(5):847-56*

This reference was deleted:  *Trotochaud K, Coleman JR, Krawiecki N, McCracken C. Moral distress in pediatric healthcare providers. J Pediatr Nurs. 2015;30(6):908-14.*

New references was inserted in 22-24:

22:	*CLSI. Collection of Diagnostic Venous Blood specimens. 7^th^ ed. CLSI standard GP41. Wayne, PA,: Clinical and Laboratory Standards Institute, 2017. Appendix A: Difficult Collection A1: Venipuncture in Children.*

23.	*World Health Organization, (2010). WHO Guidelines on Drawing Blood: Best Practices in Phlebotomy, Paediatric and neonatal blood sampling.*

24.	*Normandin PA, Benotti SA. Pediatric Phlebotomy: Taking the Bite Out of Dracula.
J Emerg Nurs. 2018 Jul;44(4):427-429. doi: 10.1016/j.jen.2018.03.017.*

Therefore, the references 25-32 was updated to 27-34 accordingly.

Additional Editor Comments:

Dear authors,

There still seem to be some major issues:

**1)** The time spent on blood collection is still an open issue. You point out that in supporting information 3a the rational for using 30 minutes as mean time for blood collections is 30 min. However, the numbers in that document read 21,3min.
Additionally, as pointed out by reviewer #1, the time seems quite long compared to other data sources. See <https://news.mayocliniclabs.com/2018/03/01/staffing-workload-phlebotomy-areas-direct-effort/>

Reply:
Thank you for valuable comment. We have now revised the time spent on blood drawings to 21.3 min and recalculated the costs accordingly.

Thank you for sending the link from Mayo Clinics, we note much shorter time on blood drawings. We have noted that the time spent on blood sampling is longer in children compared with adults. A clear distinction between blood sampling in children and adults, also stated in paediatric phlebotomy guidelines from WHO, Karolinska University Hospital Astrid Lindgren’s Children’s Hospital and CLSI Standard GP41 7^th^ Ed*, is the preparation of the child and the use of pain relief methods, this can probably explain one of the reasons of the deviation in time spent in blood drawings between paediatric and adult care. We have updated the discussion to cover this topic more thoroughly, page 13, line 229-239.

**CLSI. Collection of Diagnostic Venous Blood specimens. 7^th^ ed. CLSI standard GP41. Wayne, PA,: Clinical and Laboratory Standards Institute, 2017. Appendix A: Difficult Collection A1: Venipuncture in Children.*

**2)** The rationale for adding up costs of different professions still is not convincing.

Please explain in the text and compare to other sources so the reader can objectively interpret your numbers compared to others.

Reply: Thank you for pointing this out. We have clarified the rationale for adding up costs in the method section, page 5, line 110-116. Please, see also the revised Table 1.

Reviewers’ comments:

Reviewer’s Responses to Questions

**Comments to the Author**

1. If the authors have adequately addressed your comments raised in a previous round of review and you feel that this manuscript is now acceptable for publication, you may indicate that here to bypass the “Comments to the Author” section, enter your conflict of interest statement in the “Confidential to Editor” section, and submit your “Accept” recommendation.

Reviewer #2: All comments have been addressed

Reviewer #3: All comments have been addressed

2. Is the manuscript technically sound, and do the data support the conclusions?

The manuscript must describe a technically sound piece of scientific research with data that supports the conclusions. Experiments must have been conducted rigorously, with appropriate controls, replication, and sample sizes. The conclusions must be drawn appropriately based on the data presented.

Reviewer #2: Yes

Reviewer #3: Partly

3. Has the statistical analysis been performed appropriately and rigorously?

Reviewer #2: Yes

Reviewer #3: N/A

4. Have the authors made all data underlying the findings in their manuscript fully available?

The [PLOS Data policy](https://eur01.safelinks.protection.outlook.com/?url=http%3A%2F%2Fwww.plosone.org%2Fstatic%2Fpolicies.action%23sharing&data=05%7C01%7Chenrik.hjelmgren%40ki.se%7C932ad0ccb386446cdd3f08db6d37aa0a%7Cbff7eef1cf4b4f32be3da1dda043c05d%7C0%7C0%7C638223858072703454%7CUnknown%7CTWFpbGZsb3d8eyJWIjoiMC4wLjAwMDAiLCJQIjoiV2luMzIiLCJBTiI6Ik1haWwiLCJXVCI6Mn0%3D%7C3000%7C%7C%7C&sdata=702zdp2Nof84BMWzyWKfPhikogPeWzPgJVlAHLbitRM%3D&reserved=0) requires authors to make all data underlying the findings described in their manuscript fully available without restriction, with rare exception (please refer to the Data Availability Statement in the manuscript PDF file). The data should be provided as part of the manuscript or its supporting information, or deposited to a public repository. For example, in addition to summary statistics, the data points behind means, medians and variance measures should be available. If there are restrictions on publicly sharing data—e.g. participant privacy or use of data from a third party—those must be specified.

Reviewer #2: Yes

Reviewer #3: (No Response)

5. Is the manuscript presented in an intelligible fashion and written in standard English?

PLOS ONE does not copyedit accepted manuscripts, so the language in submitted articles must be clear, correct, and unambiguous. Any typographical or grammatical errors should be corrected at revision, so please note any specific errors here.

Reviewer #2: Yes

Reviewer #3: Yes

6. Review Comments to the Author

Please use the space provided to explain your answers to the questions above. You may also include additional comments for the author, including concerns about dual publication, research ethics, or publication ethics. (Please upload your review as an attachment if it exceeds 20,000 characters)

Reviewer #2: Thank you for addressing my comments on the first version. I have no further comments on the manuscript

Reviewer #3: I find the replies satisfying.

I do however still find that the terms blood samples and blood samplings (= drawings) are used incorrectly: The authors explain that the 54,040 refers to unique blood samplings. This is however not what the abstract says: “The annual cost of PAE was estimated to be 84,000 euros per 54,040 blood samples, which corresponds to 15,500 euros per 10,000 samples or 1.5 euros per sample.” I presume this is the cost per drawing? Please be more specific and recalculate if necessary – cost per drawing and per sample (with tube costs etc.) should be divided.

Reply: Thank you for pointing this out. We have revised accordingly and made it clearer in the text by changing to “drawings” instead of “samples/samplings” where it is appropriate. This has been done in the title, abstract and within the whole manuscript.

The cost is estimated out of different ways of drawings when taking it venous or capillary. We added a Supplementary Table 1b showing the use of how the material was used for each method.

Regarding the incorrect filling, I am well aware of the fact that incorrect filled samples can occur in all different “testings” – but the importance is not the same at all: The coagulation analyses will for certain be errorred if samples are underfilled, while most other measurements will not be affected.

Reply: Thank you once again for discussing this further. We agree with the point that coagulation errors are more sensitive for underfilled blood samples. This has now been noted in the Discussion section, page 13, line 231-233.

7. PLOS authors have the option to publish the peer review history of their article ([what does this mean?](https://eur01.safelinks.protection.outlook.com/?url=https%3A%2F%2Fjournals.plos.org%2Fplosone%2Fs%2Feditorial-and-peer-review-process%23loc-peer-review-history&data=05%7C01%7Chenrik.hjelmgren%40ki.se%7C932ad0ccb386446cdd3f08db6d37aa0a%7Cbff7eef1cf4b4f32be3da1dda043c05d%7C0%7C0%7C638223858072703454%7CUnknown%7CTWFpbGZsb3d8eyJWIjoiMC4wLjAwMDAiLCJQIjoiV2luMzIiLCJBTiI6Ik1haWwiLCJXVCI6Mn0%3D%7C3000%7C%7C%7C&sdata=auSKRPHQluaZt19TgrUFlae%2Bj4e9i0L1%2Bhd3RA81N1E%3D&reserved=0)). If published, this will include your full peer review and any attached files.


If you choose “no”, your identity will remain anonymous but your review may still be made public.


**Do you want your identity to be public for this peer review?** For information about this choice, including consent withdrawal, please see our [Privacy Policy](https://eur01.safelinks.protection.outlook.com/?url=https%3A%2F%2Fwww.plos.org%2Fprivacy-policy&data=05%7C01%7Chenrik.hjelmgren%40ki.se%7C932ad0ccb386446cdd3f08db6d37aa0a%7Cbff7eef1cf4b4f32be3da1dda043c05d%7C0%7C0%7C638223858072703454%7CUnknown%7CTWFpbGZsb3d8eyJWIjoiMC4wLjAwMDAiLCJQIjoiV2luMzIiLCJBTiI6Ik1haWwiLCJXVCI6Mn0%3D%7C3000%7C%7C%7C&sdata=NEGi0h0reLqEo1qAaMz4SvEMZJYHi%2BFAPvLfY8Tn9EA%3D&reserved=0).

Reviewer #2: No

Reviewer #3: No

[NOTE: If reviewer comments were submitted as an attachment file, they will be attached to this email and accessible via the submission site. Please log into your account, locate the manuscript record, and check for the action link "View Attachments". If this link does not appear, there are no attachment files.]

While revising your submission, please upload your figure files to the Preflight Analysis and Conversion Engine (PACE) digital diagnostic tool, [https://pacev2.apexcovantage.com/](https://eur01.safelinks.protection.outlook.com/?url=https%3A%2F%2Fpacev2.apexcovantage.com%2F&data=05%7C01%7Chenrik.hjelmgren%40ki.se%7C932ad0ccb386446cdd3f08db6d37aa0a%7Cbff7eef1cf4b4f32be3da1dda043c05d%7C0%7C0%7C638223858072703454%7CUnknown%7CTWFpbGZsb3d8eyJWIjoiMC4wLjAwMDAiLCJQIjoiV2luMzIiLCJBTiI6Ik1haWwiLCJXVCI6Mn0%3D%7C3000%7C%7C%7C&sdata=bemkt7Uw11TM%2BfvuiiQjPUy2PDTni1FJhMccq9WIf7M%3D&reserved=0). PACE helps ensure that figures meet PLOS requirements. To use PACE, you must first register as a user. Registration is free. Then, login and navigate to the UPLOAD tab, where you will find detailed instructions on how to use the tool. If you encounter any issues or have any questions when using PACE, please email PLOS at [figures@plos.org](mailto:figures@plos.org). Please note that Supporting Information files do not need this step.

*In compliance with data protection regulations, you may request that we remove your personal registration details at any time.* [*(Remove my information/details)*](https://eur01.safelinks.protection.outlook.com/?url=https%3A%2F%2Fwww.editorialmanager.com%2Fpone%2Flogin.asp%3Fa%3Dr&data=05%7C01%7Chenrik.hjelmgren%40ki.se%7C932ad0ccb386446cdd3f08db6d37aa0a%7Cbff7eef1cf4b4f32be3da1dda043c05d%7C0%7C0%7C638223858072703454%7CUnknown%7CTWFpbGZsb3d8eyJWIjoiMC4wLjAwMDAiLCJQIjoiV2luMzIiLCJBTiI6Ik1haWwiLCJXVCI6Mn0%3D%7C3000%7C%7C%7C&sdata=kBOuQ%2FHPr73YOumIKsQx1CxqwNRunFTmzDSGTTq8tFU%3D&reserved=0)*. Please contact the publication office if you have any questions.*
